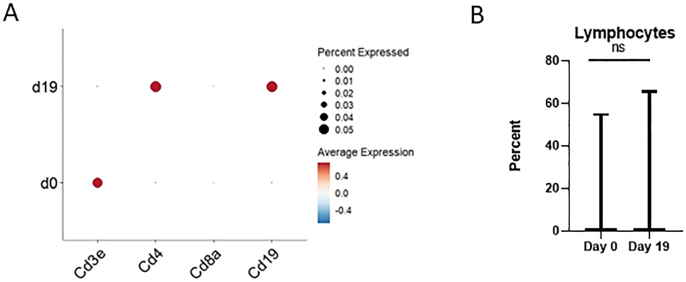# Elucidating cardiac fibroblasts heterogeneity and activation during experimental autoimmune myocarditis using spatial transcriptomics

**DOI:** 10.1016/j.bbrep.2025.102344

**Published:** 2025-11-20

**Authors:** Monika Stefańska, Katarzyna Sarad, Marta Kot, Marcin Ruciński, Martyna Strzelec, Daria Krzysztofik, Eric L. Lindberg

**Affiliations:** a Jagiellonian University Medical College, Department of Clinical Immunology, Krakow, Poland; b NanoBioMedical Centre, Adam Mickiewicz University, Poznan, Poland; c Faculty of Biophysics, Biochemistry and Biotechnology, Jagiellonian University, Krakow, Poland; d Doctoral School of Exact and Natural Sciences, Jagiellonian University, Kraków, Poland; e Jagiellonian University Medical College, Faculty of Medicine, Institute of Pediatrics, Department of Transplantation, Krakow, Poland; f Poznan University of Medical Sciences, Department of Histology and Embryology, Poznan, Poland; g Poznan University of Medical Sciences, Department of Bioinformatics and Computational Biology, Poznan, Poland; h Jagiellonian University Medical College, Doctoral School of Medical and Health Sciences, Krakow, Poland; i Małopolska Centre of Biotechnology, Jagiellonian University, Kraków, Poland; j Department of Medicine I, LMU University Hospital, Munich, Germany; k Gene Center, Department of Biochemistry, Ludwig Maximilians Universität, Munich, Germany

**Keywords:** Myocarditis, Dilated cardiomyopathy, Cardiac fibroblast, Experimental autoimmune myocarditis, Spatial transcriptomics

## Abstract

Dilated cardiomyopathy (DCM), which develops from myocardial inflammation, is frequently linked to a poor prognosis and fatal outcome. Experimental autoimmune myocarditis (EAM) serves as an animal model of CD4^+^ T cells-dependent acute myocarditis that is followed by the development of post-inflammatory DCM. The study's objective was to employ spatial transcriptomics to investigate how cardiac fibroblasts' transcriptional patterns changed during the acute inflammatory phase of EAM.

Our spatial transcriptomic analysis on 1545 cardiac fibroblasts enriched spots, in 657 of which fibroblasts were dominant cell population, resulted in identification of six distinct fibroblast subsets, which frequency changed during the acute inflammatory phase of EAM. The observed spatiotemporal colocalization of infiltrating immune cells with fibroblasts during the acute inflammatory phase may indicate that fibroblast activation is associated with, or possibly influenced by, the presence of myeloid cell infiltrates. Our findings suggest that acute myocardial inflammation may trigger a fibroblast-to-myofibroblast transition and a pro-inflammatory transcriptional response in cardiac fibroblasts. New treatment approaches for inflammatory heart disease may be developed with the aid of an understanding of the transcriptomic signatures on the spatial level.

## Highlights

• Spatial transcriptomics enables dissection of cardiac fibroblasts' heterogeneity during experimental autoimmune myocarditis.
• Cardiac fibroblasts heterogeneity alters during acute phase of EAM.
• Cardiac fibroblast and myeloid inflammatory cells spatiotemporally colocalize during the progression of EAM.

## 1 Introduction

Cardiomyopathy, which develops directly from myocardial inflammation, is linked to a poor prognosis for most patients. Autoimmunity, bacterial, viral, including COVID-19, protozoal infections as well as drugs, hazardous substances are the etiological backgrounds of myocarditis [1]. The etiopathogenesis of the disease plays a pivotal role in determining its severity and progression.

Fibroblasts and immune cells play crucial roles in the development of myocarditis, and also possibly in its progression. During inflammation, fibroblasts differentiate into myofibroblasts, which secrete extracellular matrix proteins to maintain tissue integrity. However, they also contribute to immune cell recruitment, potentially exacerbating disease severity. Additionally, macrophages have been identified as key factors influencing cardiac remodelling and fibrosis [2,3].

The purpose of this study was to evaluate the transcriptomic signature of cardiac fibroblasts’ activation and inflammatory response at the acute inflammatory phase of EAM utilising spatial transcriptomics. In this study, we aimed to characterize the dynamic alterations in the expression of selected factors associated with proinflammatory cytokines and receptors, inflammatory-related pathways across distinct subpopulations of cardiac fibroblasts as well as define cellular interactions between fibroblasts and immune cells. By integrating these molecular insights, we sought to provide a characterization of the transcriptional landscape underlying the pathophysiological remodelling during the acute phase of EAM.

## 2 Materials and methods

### 2.1 Mice

Wild type BALB/c mice were housed under standard conditions (12h light/dark cycle, 21 ± 1 °C, 45–55 % humidity) with free access to food and water. Experiments complied with Polish law and were approved by the Local Ethical Committee in Kraków (approval number: 465/2021, 586/2021) following EU Directive 2010/63/EU.

### 2.2 EAM induction

EAM was induced in 6–8 weeks old male mice (n = 8 per group) by subcutaneous injection of α-MyHC 614–634 peptide (Ac-RSLKLMATLFSTYASADR-OH, Caslo, Lyngby, Denmark) in dose of 200 μg emulsified 1:1 with Complete Freund's Adjuvant at day 0 and 7. Upon completion of the experiments, the mice were euthanized *via* cervical dislocation.

### 2.3 Spatial transcriptomics

Representative heart samples at acute inflammatory stage of experimental autoimmune myocarditis (day 19) as well as healthy controls (day 0), previously perfused with PBS, were embedded and frozen in OCT buffer (Merck), by simultaneous immersion in Isopentane (Supelco) and liquid nitrogen (n = 8 per group). Before proceeding to Visium spatial transcriptomics assay RNA integrity number (RIN) was determined. Samples with RIN below 7 were excluded from further analysis. Spatial transcriptomics assay was performed using Visium 10x Genomics technology (PN-1000187) following manufacturers' recommendations on n = 1 slide per condition. Imaging was performed using an IX70 microscope (Olympus Corporation, Japan) and CellSensDimension software. Spatial transcriptomics cDNA libraries were performed following manufacturers’ recommendations (CG000239 RevF, 10x Genomics), dual indexed and pair-end sequenced 28-10-10-90 cycles on Illumina Novaseq6000 system.

### 2.4 Spatial RNA-sequencing data processing

FASTQ files were mapped to the mouse genome (mm10, 2020-A) using SpaceRanger v2.0.1. Filtered feature-barcode matrices were loaded into Seurat v4.3.0 with Load10X_Spatial, extracting gene expression and tissue images. Quality control kept spots with >500 UMIs and >300 genes. Data normalization and variance stabilization used SCTransform [4], which performs regularized negative binomial regression to remove technical noise while highlighting biological variation. Processed data stored in the “SCT” slot of the Seurat object was used for all downstream analyses. Dimensionality reduction was done with PCA (RunPCA), neighbors identified with FindNeighbours, and clustering via FindClusters, following Seurat guidelines. Visualization used SpatialFeaturePlot, UMAP, and packages like ggplot2 and dplyr. Differential expression was analyzed with FindMarkers using the Wilcoxon test (log2FC > 0.25). The deconvolution analysis was carried out using the CARD package, following the methodology described in the preprint manuscript [5]. LIANA analysis was performed following the methodology described in Ref. [6]. Colocalization analysis was performed using the Squidpy framework (Python toolkit) [7]. A spatial neighborhood graph was built with squidpy's sq.gr.spatial_neighbors using Delaunay triangulation on 2D spatial coordinates, producing an undirected graph connecting nearby points without radius/k parameters. Colocalization analysis used a permutation approach correlating neighborhood-averaged geneB to geneA expression via this adjacency. Statistical significance was tested by shuffling geneB expression 100 times, calculating empirical p-values.

Visium n = 1 slide/condition; statistics are exploratory spot-level summaries.

## 3 Results and discussion

### 3.1 Spatial transcriptomics reveal the molecular changes in experimental autoimmune myocarditis

**Fig. 1:**
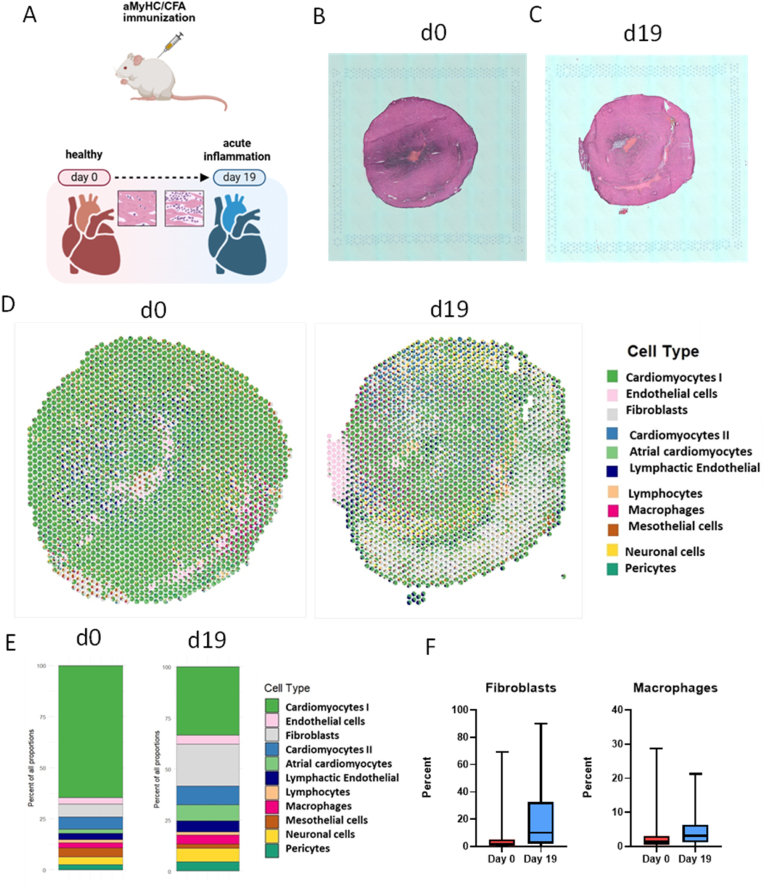
**Spatial transcriptomics revealed the presence of different cell types of cardiac fibroblasts during EAM**. **A:** Schematic representation of the experimental layout.**B**: Microscopy image showing tissue at day 0 of EAM (healthy heart) on Visium 10x Genomics slide, haematoxylin and eosin stain, 20× magnification. **C:** Microscopy image showing tissue at day 19 of EAM (acute inflammation) on Visium 10x Genomics slide, haematoxylin and eosin stain, 20× magnification. **D:** SpatialPlot of murine heart at day 0 and 19 following deconvolution shows the contribution of each cell type into each spot. **E:** Bar plot showing the frequency of different cell types at baseline (healthy heart – day 0) and acute inflammatory phase of EAM (day 19). **F:** BoxPlot showing the contribution of Cardiac Fibroblasts and Macrophages into spots on Visium slide following spatial deconvolution at day 0 and day 19. Visium n = 1 slide/condition; statistics are exploratory spot-level summaries.

Experimental autoimmune myocarditis (EAM) was induced in susceptible BALB/c mice. The hearts obtained according to the protocol schematically depicted in Fig. 1A, were evaluated based on haematoxylin-eosin staining and microscopic observation. As shown in Fig. 1B, C the presence of infiltrating myeloid cells is evident during the acute inflammatory phase (day 19). As opposed to day 0, where the number of myeloid cells is much smaller, indicating minimal baseline infiltration. Microscopy images provided also information about increased size of the heart which suggest autoimmune-related heart remodelling (Fig. 1B and C).

Spatial transcriptomics analysis of 3789 spots obtained from representative hearts of different stages of experimental autoimmune myocarditis (day 0 – healthy heart (n = 1), day 19 – acute inflammation (n = 1)), proceeded by spatial deconvolution using CARD method revealed the presence of 11 distinct clusters of spots rich in cells with different transcriptomic signatures (Fig. 1D). The frequency of each cell type following deconvolution is depicted in Fig. 1E. Due to the resolution of the method and the complexity of the tissue it was not possible to annotate spots to certain cell type as there is usually between one to four cells present per spot. However, the obtained clusters of spots were successfully described in the context of enriched fibroblast- and immune-related transcripts. The percentage of fibroblasts and macrophages within Visium spots was quantified by deconvolution and is shown in Fig. 1F. There was a notable increase in the proportion of fibroblasts at day 19 compared to day 0, as indicated by the boxplot analysis. Similarly, the percentage of macrophages was markedly higher at day 19 versus day 0. These results demonstrate dynamic changes in cellular composition within the spots over time, with both fibroblast and macrophage populations expanding by day 19 after the EAM induction (Fig. 1F). As EAM is also driven by T-lymphocytes, we have checked the expression of lymphocytes specific marker genes. As shown in Supplementary Fig. 1A, there is an upregulation of lymphocytes specific genes such as Cd4, Cd8a and Cd19 at day 19 following EAM induction. Unfortunately, no significant differences were observed when compared percent of spots occupied by lymphocytes at both experimental timepoints (Supp.Fig. 1B). Given the low number of lymphocyte-dominant spots, a statistically powered proximity test for lymphocytes was not feasible. As such, claims regarding lymphocyte spatial distribution are strictly descriptive.

### 3.2 Cardiac fibroblasts in experimental autoimmune myocarditis (EAM)

Fibroblasts are crucial components of the mouse heart. These cells maintain cardiac tissue integrity by secreting extracellular matrix and engaging in cell-to-cell communication to preserve tissue homeostasis [8]. Cardiac fibroblasts contribute to heart disease by promoting fibrosis and inflammation, working closely with infiltrating myeloid cells. The genesis of inflammatory heart disease is most likely centred on the proinflammatory function of cardiac fibroblasts [[8], [9], [10]].

**Fig. 2:**
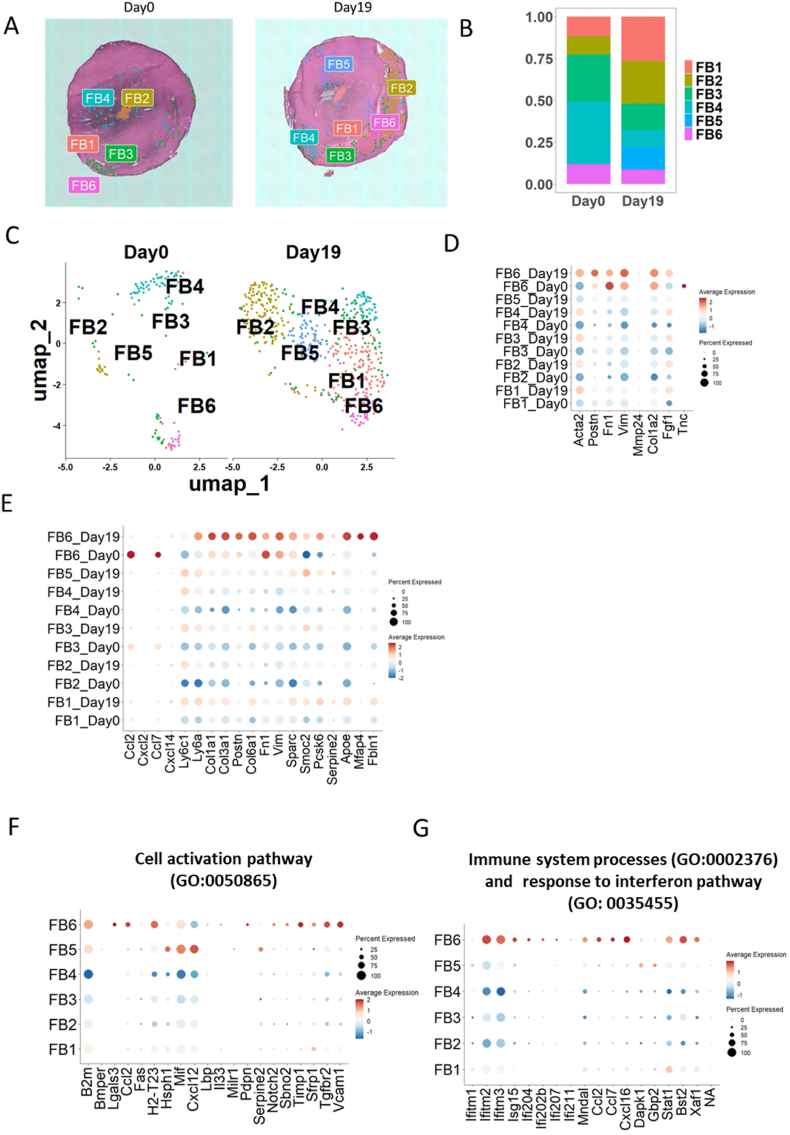
**Spatial transcriptomics revealed the presence of different subsets of cardiac fibroblasts during experimental autoimmune myocarditis**. **A:** SpatialPlot of murine heart at day 0 and 19 shows the presence of 6 distinct subsets of cardiac fibroblasts FB1-FB6. **B:** Bar plot showing the frequency of different fibroblast-subsets at different stages of EAM **C:** UMAP clustering of cardiac fibroblasts from murine heart at different stages of EAM (day 0 and day 19) shows the presence of different subsets of cardiac fibroblasts. **D:** Dot-plot showing the expression of 8 pro-fibrotic genes within fibroblast-subset at different stages of EAM (day 0 – healthy heart, day 19 acute inflammation). **E:** Dot-plot showing the expression of selected genes within fibroblast-subset at different stages of EAM (day 0 – healthy heart, day 19 acute inflammation). **F:** Dot-plot showing the expression of selected genes related with cell activation pathway (GO:0050865) within fibroblast-subset at healthy heart (day 0) and during acute inflammatory phase (day 19). **G:.** Dot-plot showing the expression of selected genes related with immune system processes (GO:0002376) and response to interferon pathway (GO:0035455) within fibroblast-subsets at healthy heart (day 0) and during acute inflammatory phase (day 19). For each group n = 1.

A detailed analysis of the transcriptomic signature of cardiac fibroblasts, performed on 657 spots where fibroblasts' were dominant cell population (136 spots at day 0 and 521 of spots at day 19), resulted in identification of six different subsets: FB1, FB2, FB3, FB4, FB5 and FB6 (Fig. 2A). At day 0, only fibroblast subsets FB1, FB2, FB3, FB4, and FB6 have been observed in heart tissue. However, during the acute inflammatory phase, the heart tissue consists of all six fibroblast subsets (FB1–FB6), indicating an expansion of fibroblast diversity in response to inflammation (Fig. 2A). The distribution of fibroblast subsets was confirmed through UMAP analysis (Fig. 2C), which revealed distinct clustering patterns between day 0 and day 19. At day 19, unique fibroblast-enriched regions corresponding to FB5 subset was identified whereas FB4 was predominantly localized at day 0 in the healthy heart, indicating differential subset dynamics during inflammation (Fig. 2C). The frequency of all cluster's changes during the time course of EAM. For instance, the most numerous cluster FB1 and FB2 expanded during the time course of EAM (Fig. 2B). Of note, cluster FB5 is only transiently present at day 19 in the acute phase of inflammation (Fig. 2B). It is worth noticing that FB4 subset which is present mostly in the healthy mouse heart and it is frequency decreasing at day 19 (Fig. 2B and C).

### 3.3 Profibrotic and proinflammatory changes across spots rich in various fibroblasts subsets

Given the pivotal role of myofibroblasts in initiating inflammatory reactions and possibly facilitating the induction of disease towards the dilated cardiomyopathy phenotype, it is important to assess the expression of a set of profibrotic genes in various locations enriched with distinct fibroblast subsets within this context. Interestingly, among all the profibrotic genes whose expression was tested: *Acta2*, *Postn*, *Fn1*, *Vim*, *Fgf1*, *Col1a2*, *Mmp24* and *Tnc*, only *Acta2* and *Fgf1* were upregulated in most of the tested spots rich in fibroblasts subsets but only at day 19 (Fig. 2D). The strongest expression of pro-fibrotic genes tested was observed in spots rich in fibroblast-subset FB6 (Fig. 2D). Interestingly, all the other pro-fibrotic genes tested were downregulated in these spots rich in fibroblasts at tested time points (Fig. 2D). The only exceptions tested is *Col1a2*, which is upregulated in FB1 subset at day 19 (Fig. 2D). Moreover, we investigated the expression of set of genes related to induction of myofibroblastic signature and disease induction in these fibroblast subsets (Fig. 2E). This group includes genes encoding various collagens, *Apoe*, *Smoc2*, and serpins which have been shown to be involved in the initiation and progression of autoimmune myocarditis in this animal model. Interestingly, the FB5 and FB6 subsets at day 19 show high expression of all the tested genes. The FB1 subset switches on the expression of majority of tested genes during the disease induction (downregulated at day 0, upregulated at day 19). In contrast, the FB4 subset strongly upregulates the expression of *Smoc2* during the myocarditis induction (Fig. 2E). These data shed the new light on the involvement of different fibroblast subsets in the induction of autoimmune myocarditis in the EAM model. Our spatial transcriptomic results show a myofibroblastic subsets are mostly present at day 19 of inflammation. *Acta2* is currently the strongest indicator of myofibroblasts and it is expressed at higher levels in the FB6 region. Recently, the *Smoc2* gene has been shown to **mediate cell-matrix interactions and facilitate fibrosis, as it is involved in the fibro-myofibroblast transition** [11]. *Smoc2* shows the highest expression at day 19 following EAM induction, mostly in spots rich in fibroblasts subsets FB5 and FB6 (Fig. 2E).

### 3.4 Fibroblast-subsets show different transcriptomic signatures and are involved in different signalling pathways

Next, we tested the involvement of different fibroblast subsets in cell signaling pathways mostly related with cell activation, interferon pathway and immune system processes Firstly, we studied further the most numerous at the acute inflammatory phase fibroblast subset FB1. This subset activated expression of genes related to cell activation pathway (GO: 0050865) such as *B2m*, *Mif*, *Cxcl12* and *Serpine2* (Fig. 2F). Proinflammatory cytokines genes related to immune system processes (GO: 0002376) and genes related to response to interferon pathway (GO: 0035455) were also expressed at the high level in this fibroblast subset (Fig. 2G). FB2 subset is not characterized by higher expression of proinflammatory cytokines and their receptors with the exception of moderate expression of *Cxcl12* (Fig. 2G) Genes related to immune system processes (GO: 0002376), response to the interferon pathway (GO: 0035455) or cell activation pathway (GO: 0050865) (Fig. 2F and G) were also downregulated in this fibroblast subset. Next, we evaluated the transcriptomic signature of the spots rich in fibroblast subset FB3 and FB4. Non-inflammatory character of FB3 and FB4 subsets is reflected in its relatively low expression level of proinflammatory cytokines and their receptors. (Fig. 2F). These subsets downregulate genes related to cell activation pathway (GO: 0050865) immune system processes (GO:0002376) and genes related to response to interferon pathway (GO: 0035455) (Fig. 2F and G).

Subset FB5 strongly upregulates genes related to cell activation pathway (GO: 0050865) and also some genes such as *Dapk1*, *Gbp2* and *Ifitm1* related to immune system processes (GO: 0002376) and to interferon (Fig. 2F and G).

The most transcriptionally active subset of fibroblasts which strongly upregulates most of the genes related to cell activation pathway (GO:0050865) as well as immune system processes (GO:0002376) and response to interferon pathway (GO:0035455) is subset FB6 (Fig. 2F and G).

### 3.5 Infiltrating immune cells and cardiac fibroblasts spatiotemporally colocalize during the acute phase of EAM

**Fig. 3:**
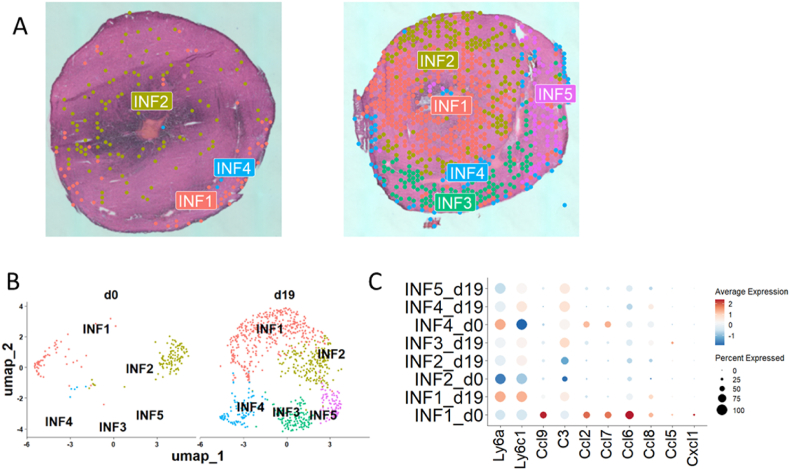
**Spatial transcriptomics revealed the presence of different subsets of inflammatory cells during EAM**. **A:** SpatialPlots showing the contribution of distinct inflammatory cells subsets during EAM – day 0 (left) and day 19 (right) **B:** UMAP clustering of inflammatory cells from murine heart at different stages of EAM (day 0 and day 19) shows the presence of different subsets of infiltrating immune cells. **C:** Dot-plot showing the expression of 10 myeloid associated genes within inflammatory cells subsets at different stages of EAM (day 0 – healthy heart, day 19 – acute inflammation). For each group n = 1.

A detailed analysis of the transcriptomic signature of infiltrating immune cells using spatial transcriptomics, performed on 1032 spots rich in infiltrating immune cells (169 spots at day 0 and 863 spots at day 19), resulted in the separation of five different subsets of inflammatory cells such as: INF1, INF2, INF3, INF4, and INF5 (Fig. 3A and B). The presence of subsets INF1, INF2 and INF4 has been observed at day 0 (healthy heart), whereas during the acute inflammatory phase, the heart tissue is composed of inflammatory cell subsets INF1–INF5 (Fig. 3A and B). Cell subset distribution has been confirmed by the UMAP plot, where it is evident that at day 19, unique immune cell-rich spots INF3, INF4 and INF5 emerge, whereas the INF2 subset is resident and present at both stages of the disease (Fig. 3A and B). The INF1 subset increases in frequency with very few spots expressing the genes typical for this subset at day 0 and it became more numerous at day 19 (Fig. 3A and B). Next, we checked the expression of genes associated with myeloid proinflammatory signature in these inflammatory subsets. Interestingly, the INF1 subset upregulates the expression of *Ly6a* and *Ly6c1* during disease development. The INF4 subset upregulates the expression of *Ly6c1* while simultaneously downregulating the expression of *Ly6a*. The complement gene *C3* is activated only during the acute inflammatory phase in all subsets except INF2 (Fig. 3C). Surprisingly, on day zero, more pro-inflammatory cytokines are secreted for INF1 compared to day 19. However, by day 19, there is an increase in *Ly6a* and *Ly6c*, suggesting a distinct activation mechanism for both the INF1 cells themselves and the cells they interact with (Fig. 3C). Our spatial transcriptomic results show a myofibroblastic subset mostly present at day 19 of inflammation. Interestingly, this subgroup colocalizes with inflammatory cells populations producing INF4 subset in space and time, which may indicate that these cell populations cooperate to worsen inflammation and initiate fibrosis.

### 3.6 Fibroblast–macrophage crosstalk dynamics revealed by ligand–receptor interactions and colocalization analysis during the acute phase of EAM

**Fig. 4:**
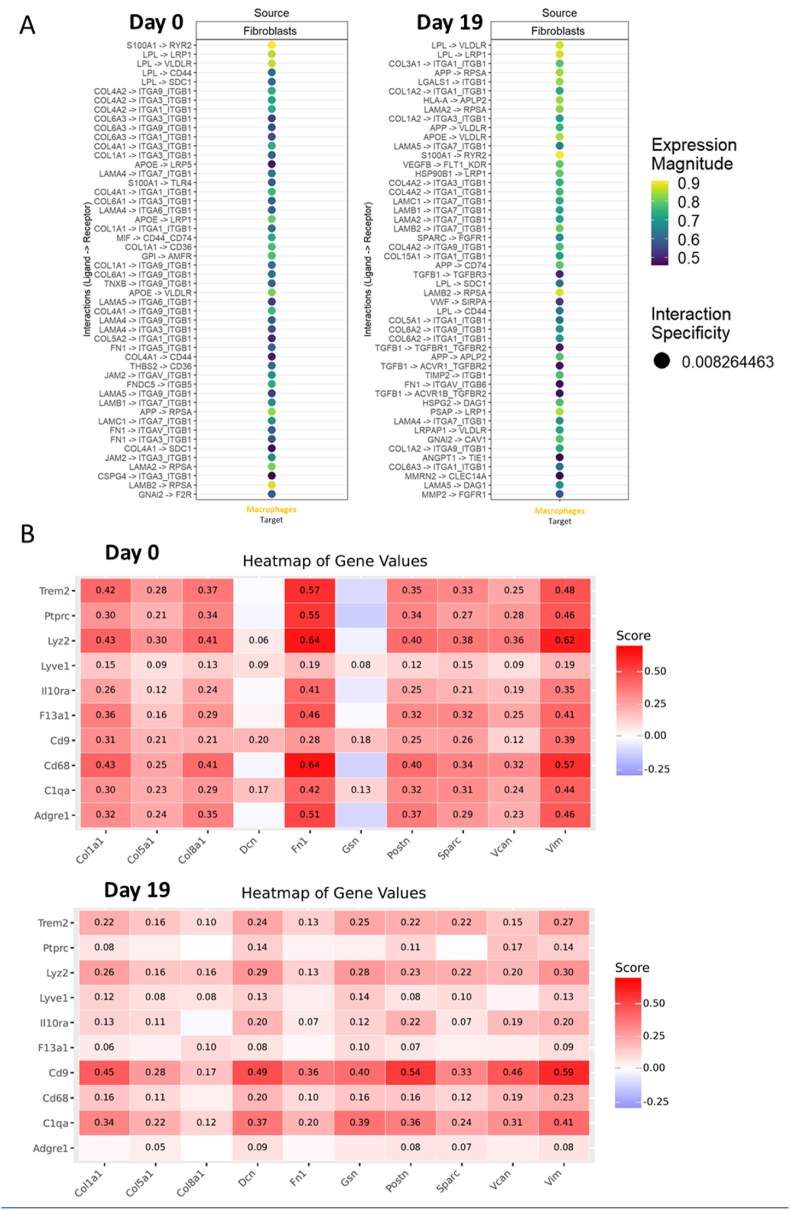
**Fibroblast–Macrophage Crosstalk Dynamics Revealed by Ligand–Receptor Interactions and Colocalization Analysis during acute phase of EAM**. **A:** Ligand–receptor interaction analysis (LIANA): an overview of ligand–receptor interactions predicted between fibroblasts (source) and macrophages (target) at day 0 and day 19. The lists show specific ligand–receptor pairs, sorted by source cell type and directionality. Dot color represents the magnitude of expression of the interacting ligand and receptor, while dot size indicates interaction specificity according to the LIANA analysis. **B:** Colocalization analysis of gene pair values. The heatmaps display colocalization scores for macrophage-associated genes (rows) and fibroblast-associated genes (columns) at day 0 and day 19. Significance was calculated using a permutation test. All depicted values have been prefiltered for p-value of 0.05. For each group n = 1.

Next, we wanted to investigate the ligand-receptor analysis to investigate the potential possible pathways at which cardiac fibroblasts communicate with macrophages which can explain the transcriptional changes in those cells between healthy heart (day 0) and acute phase of inflammation (day 19). As shown in Fig. 4A, at day 0, fibroblasts communicate with macrophages by S100A1-RYR2, LPL-LRP1, LAMB2 – RPSA pathways. These interactions likely contribute to maintaining tissue homeostasis by regulating calcium signalling, lipid metabolism, and extracellular matrix remodelling. Specifically, the S100A1–RYR2 axis may modulate intracellular calcium dynamics crucial for cellular function, while the LPL–LRP1 pathway could influence lipid handling and immune regulation. The interaction between LAMB2 and RPSA suggests a role in mediating cell adhesion and matrix-immune cell crosstalk. Together, these pathways highlight complex and coordinated communication between fibroblasts and macrophages that support the physiological state of the heart (Fig. 4A). In contrast, at day 19 the scope of interactions is slightly different and pathways with the strongest expression magnitude include: APOE-VLDLR, LGALS1-ITGB1 and also LAMB2, RPSA. The communication via COL–ITGA/B pathway is much stronger at day 19 when compared to day 0 (Fig. 4A). The pronounced upregulation of the COL–ITGA/B pathway at this stage indicates enhanced extracellular matrix interactions likely contributing to inflammatory remodelling processes. These changes reflect an adaptive shift in intercellular signalling that may underlie the transcriptional reprogramming observed in both fibroblasts and macrophages during cardiac inflammation.

To assess the interaction between key genetic markers of fibroblasts and myeloid cells at day 0 and day 19, we performed colocalization analysis. This analysis calculated enrichment scores reflecting whether one gene is enriched at locations with the highest expression of the other gene. As shown in Fig. 4B and Supplementary Figs. 2 and 3, the majority of fibroblast marker genes colocalize with established myeloid cell markers. Notably, the enrichment scores of these colocalizations vary between the healthy state and the acute inflammatory phase, with genes such as *Gsn* and *Dcn* showing increased colocalization with myeloid cells specifically at day 19 (Fig. 4B). These findings underscore dynamic spatial interactions between fibroblasts and myeloid cells during inflammation, suggesting coordinated cellular crosstalk that may contribute to disease progression.

### 3.7 Translational comparison with viral myocarditis models

To further expand the relevance of our findings in the context of infectious myocarditis, we provide a comparative perspective, referencing recent spatial transcriptomics studies on viral models: reovirus-induced [12] and coxsackievirus B3-induced fulminant myocarditis [13]. Both viral models, analyzed with spatial and single-cell transcriptomics, reveal pronounced spatial heterogeneity of myocardial injury, dense infiltration by immune cells (notably T cells and macrophages), and strong upregulation of interferon-related antiviral pathways. Notably, viral myocarditis is characterized by the presence of infected cardiomyocytes and high interferon-γ activity, as well as injury signatures localized to specific regions adjoining sites of viral replication and endothelial infection.

In contrast, our autoimmune (EAM) model recapitulates immune-driven tissue damage and fibroblast activation, but lacks the direct cytopathic effects and robust early interferon signatures seen in viral myocarditis. However, both models demonstrate pivotal roles for myeloid–fibroblast communication and patchy spatial patterns of injury, underscoring shared pathways in tissue remodelling. Importantly, recent results from Li et al. highlight the IFN-γ/Spi1 axis and mesothelial infection as unique drivers in fulminant viral myocarditis, with translational implications for immunomodulatory therapies [13]. These comparisons improve understanding of our findings. While single-disease models reveal common inflammation and remodelling mechanisms, applying them directly to infectious myocarditis requires caution. Future research will incorporate pathogen-induced models and spatial profiling to validate these results.

## 4 Conclusions

In this study, we have discussed various fibroblast subsets during the acute phase of EAM (day 19) in comparison to healthy heart (day 0). We have highlighted the potential pathways through which myeloid cell infiltration coincides with and may influence activation of cardiac fibroblasts. We have identified signalling pathways that can potentially explain changes observed in fibroblasts transcriptomic signature during the acute inflammatory phase of EAM. This may lead to the development of novel cell subtype-specific therapies or, more importantly, diagnostic tools for the accurate diagnosis, targeting, and monitoring of the onset of cardiac inflammation, potentially leading to fibrosis.

## Data availability statement

Data are available in a public, open access repository Gene Expression Omnibus (https://www.ncbi.nlm.nih.gov/geo/) with the accession number GSE248347.

## Funding

This research was funded by the 10.13039/501100004281National Science Centre (Poland), grant number 2022/06/X/NZ5/00010 (to Monika Stefańska) and 2023/51/D/NZ7/00609 (to Monika Stefańska)

## CRediT authorship contribution statement

**Monika Stefańska:** Conceptualization, Data curation, Formal analysis, Funding acquisition, Investigation, Methodology, Project administration, Software, Supervision, Visualization, Writing – original draft, Writing – review & editing. **Katarzyna Sarad:** Investigation, Software, Visualization, Writing – original draft, Writing – review & editing. **Marta Kot:** Investigation, Visualization, Writing – original draft, Writing – review & editing. **Marcin Ruciński:** Writing – review & editing, Formal analysis, Software, Visualization. **Martyna Strzelec:** Investigation, Writing – original draft. **Daria Krzysztofik:** Investigation, Visualization, Writing – original draft. **Eric L. Lindberg:** Writing – review & editing, Formal analysis, Software, Visualization.

## Declaration of competing interest

The authors declare that they have no known competing financial interests or personal relationships that could have appeared to influence the work reported in this paper.

## Figures and Tables

**figs1:**