# Supplementary figures and images for: Elucidating cardiac fibroblasts heterogeneity and activation during experimental autoimmune myocarditis using spatial transcriptomics
Source: Biochem Biophys Rep. 2025 Nov 20;44:102344. doi: 10.1016/j.bbrep.2025.102344 (PMC12670445; doi:10.1016/j.bbrep.2025.102344)

### Heatmap of Gene Values

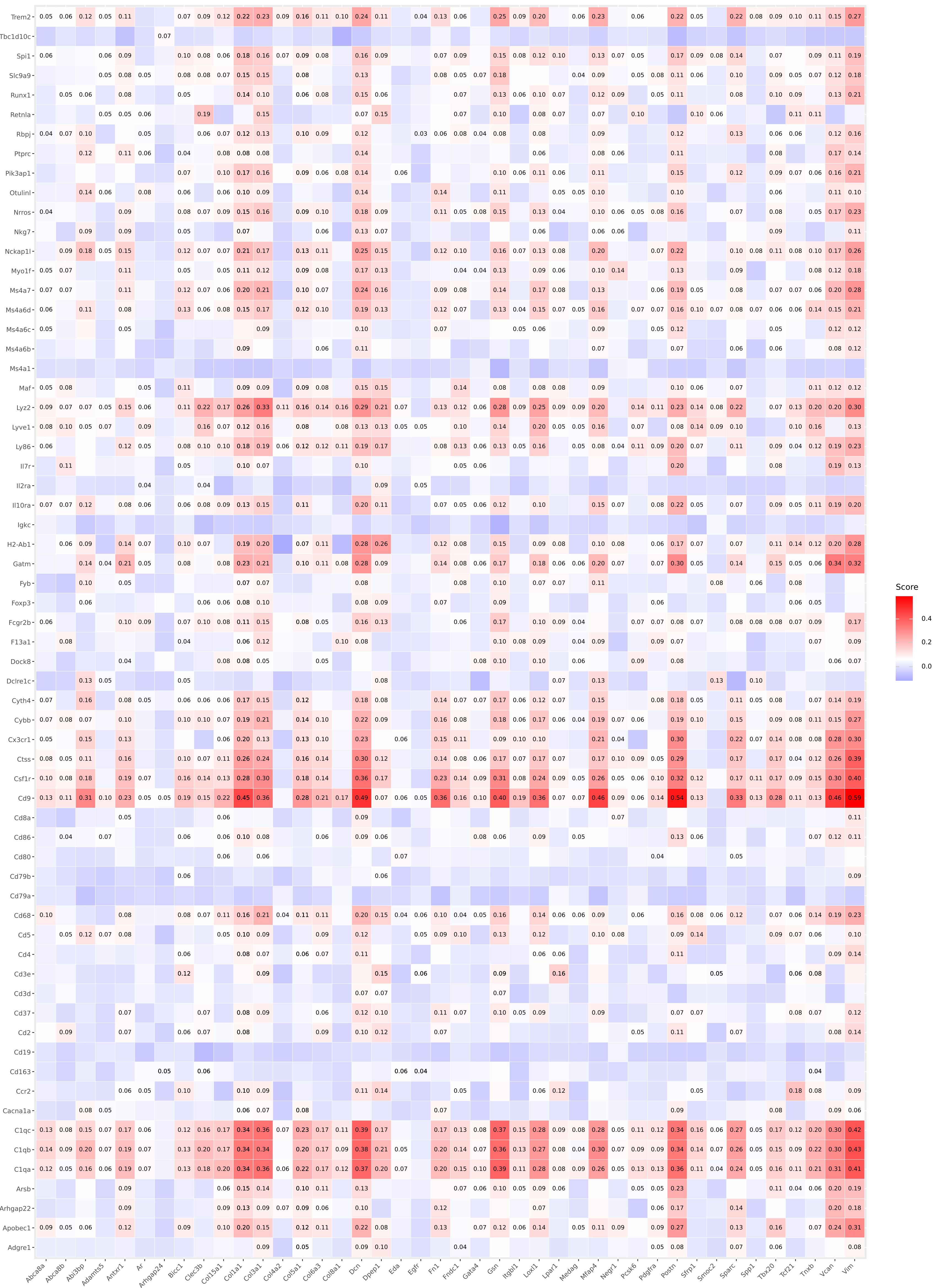

Supplement: Multimedia component 9 [file mmc9.pdf]

### Heatmap of Gene Values

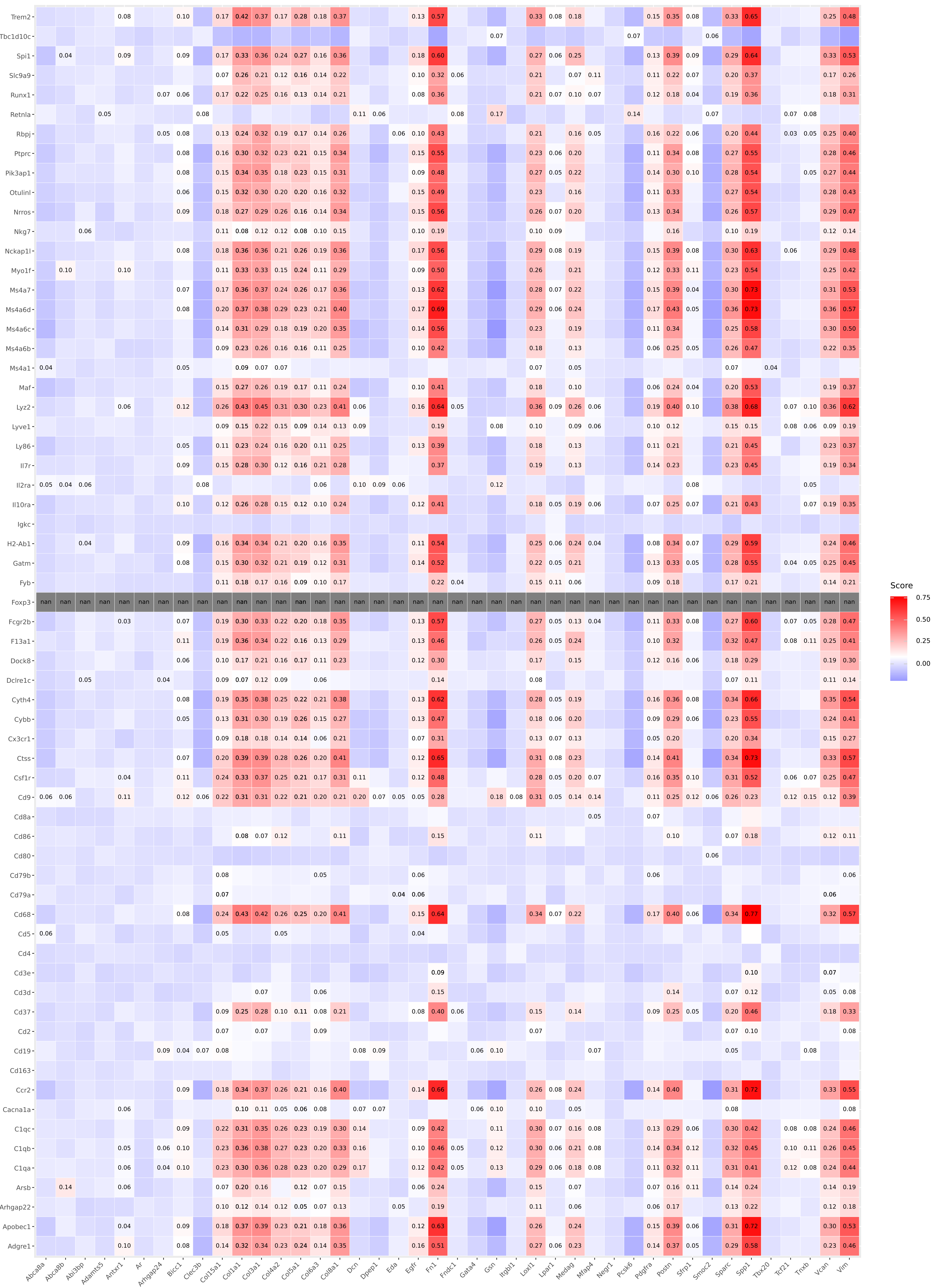

Supplement: Multimedia component 10 [file mmc10.pdf]
